# A laparoscopic approach to address massive splenomegaly, symptomatic cholelithiasis, and a planned postoperative pregnancy: A case report

**DOI:** 10.1002/ccr3.6831

**Published:** 2023-01-23

**Authors:** Alexandra A. Bishop, Eric Krohn, Victor R. Vakayil, Kyle Pribyl, Mark T. Reding, Christopher Tignanelli, James V. Harmon

**Affiliations:** ^1^ University of Minnesota Medical School Minneapolis Minnesota USA; ^2^ Department of Surgery University of Minnesota Minneapolis Minnesota USA; ^3^ Department of Medicine University of Minnesota Minneapolis Minnesota USA

**Keywords:** acute medicine, allergy and immunology, case report, general surgery, hematology, obstetrics and gynecology

## Abstract

We report long‐term follow‐up of a patient who underwent a tailored laparoscopic procedure for symptomatic cholelithiasis, massive splenomegaly, and a planned pregnancy. There were no complications, and the patient remained symptom‐free at the 5‐year follow‐up. We supplemented our case report with national surgical data demonstrating the safety of laparoscopic splenectomy.

## Abstract 2

Laparoscopic splenectomy is a safe and effective approach for patients with immune thrombocytopenic purpura, even those with massive splenomegaly. We highlight the benefits of tailoring the surgical approach to address the patient's surgical needs.

## 1 INTRODUCTION

We aimed to demonstrate that surgery can be tailored to address a patient's unique medical and surgical issues. Our patient presented with severe symptomatic cholelithiasis and massive splenomegaly; she had additionally planned for pregnancy shortly after surgery. A combined laparoscopic cholecystectomy and splenectomy was proposed to avoid large abdominal incisions immediately prior to the planned pregnancy. No procedural complications were observed, and the patient successfully carried a healthy pregnancy to term within 1 year of surgery. We have provided supplemental data from the National Surgical Quality Improvement Program (NSQIP) database that compares the complication rates between laparoscopic splenectomy (LS) and open splenectomy (OS) for thrombocytopenia. These data support the safety and efficacy of LS.

We discovered two related studies on splenectomy for thrombocytopenia from the literature. A NSQIP study published in 2013 compared patients who had undergone LS and OS and were cared for prior to 2010. A meta‐analysis published in 2021 summarized the data from smaller reported series.^1^, ^2^ This case report and NSQIP study compared the expected complications and patient outcomes for LS and OS in this era, where LS is the standard approach. This study demonstrates the value of a tailored surgical approach. In addition, our supplemental NSQIP data reinforce the safety of LS. The patient's excellent clinical outcome demonstrates that combined laparoscopic cholecystectomy and splenectomy is safe, even in patients planning for pregnancy shortly after surgery. This case report has been reported in line with the SCARE Criteria.^3^

### 1.1 Case history

We report the 5‐year follow‐up of a 28‐year‐old Caucasian woman who underwent combined laparoscopic cholecystectomy and splenectomy for severe symptomatic cholelithiasis and massive splenomegaly; she had also planned for pregnancy shortly after surgery.

**FIGURE 1:**
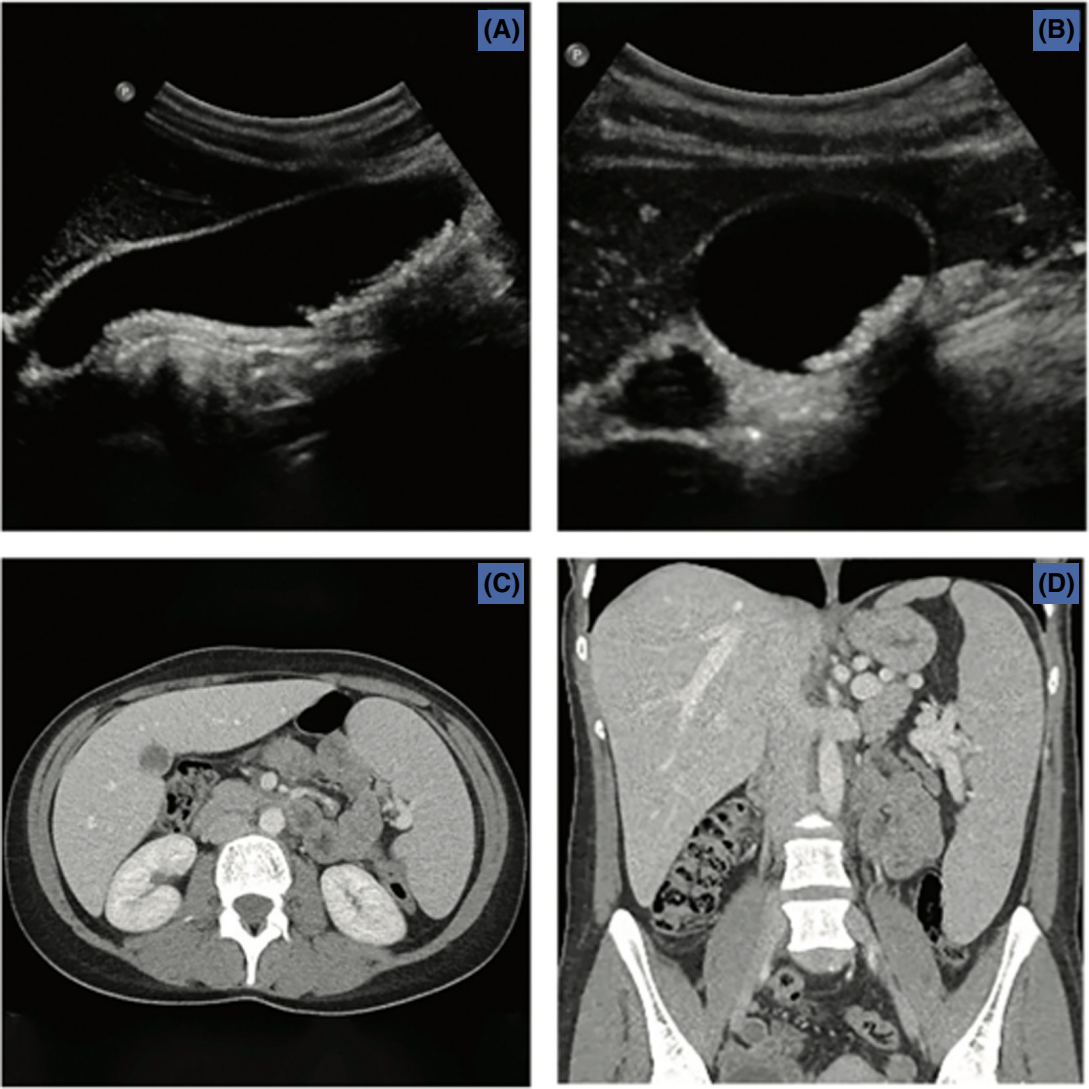
Imaging Studies (A) Ultrasound long‐axis view—cholelithiasis; (B) Ultrasound short‐axis view—cholelithiasis; (C) Abdominal computed tomography (CT) transverse—splenomegaly; (D) Abdominal CT coronal—massive splenomegaly

The patient presented with recurrent, severe postprandial biliary colic, nausea, and vomiting, which were associated with multiple gallstones on ultrasound, as demonstrated in Figure 1A,B. The initial workup for cholelithiasis revealed moderate thrombocytopenia (range: 50–60 × 10^3^/μl) and mild anemia, which was evaluated by the Hematology Division. Additionally, CA 19‐9 and CA 125 levels, a peripheral smear, and an abdominal computed tomography scan (CT abdomen) were obtained. The tumor markers were found to be significantly elevated, with a CA 19‐9 of 92 units/ml (normal range: 0–37 units/ml) and CA 125 of 818 units/ml (normal range: 1–30 units/ml). The peripheral smear showed moderate thrombocytopenia with normal platelet morphology, and a mild normocytic normochromic anemia with increased erythrocyte regeneration. There was no evidence of hemolysis. A bone marrow biopsy was planned for a platelet count below 50 × 10^3^/μl; however, the platelet count did not decrease to this level. The CT abdomen showed massive splenomegaly (22 cm × 14 cm × 6 cm), as demonstrated in Figure 1C,D. The patient's presentation and laboratory data excluded Hemolysis, Elevated Liver enzymes, and Low Platelets (HELLP) syndrome, thrombotic thrombocytopenic purpura, disseminated intravascular coagulation, and antiphospholipid antibody syndrome from the differential diagnosis. There was no clinical evidence of hepatitis C, human immunodeficiency virus, systemic lupus erythematous, or thyroid disease. Although unconfirmed, immune thrombocytopenic purpura (ITP) could not be excluded. Steroids, thrombopoietin (TPO) receptor agonists, and rituximab were not initiated, as the patient's platelet count did not decrease below 50 × 10^3^/μl.

There is no effective medical therapy for symptomatic cholelithiasis or massive splenomegaly; therefore, surgery is considered the best option. Moreover, splenectomy was indicated for our patient due to the life‐threatening risk of splenic rupture and rare possibility of lymphoma, owing to the markedly elevated tumor markers. Massive splenomegaly can be a sign of primary splenic or other lymphomas, and a splenectomy is required to confirm the diagnosis in the absence of another option for tissue biopsy.^4^ Furthermore, CA 125 has emerged as a tumor marker for lymphoma. A case report of a patient with a history of ovarian cancer specifically linked elevated CA 125 levels to the patient's new follicular lymphoma diagnosis, rather than to ovarian cancer.^5^

Our patient underwent combined laparoscopic cholecystectomy for symptomatic cholelithiasis and splenectomy for massive splenomegaly. The surgery was performed by the staff surgeon with the assistance of a chief resident. The meningococcal, Haemophilus influenzae type B, and Pneumovax 23 vaccines were administered 2 weeks prior to surgery. The procedure was performed in the partial right lateral position. Five laparoscopic ports were placed, and the laparoscopic LigaSure™ device (Medtronic) was utilized. The splenic artery was ligated using Hem‐O‐Lok clips, and the splenic vein was divided using the endo GIA stapler (Ethicon). Two laparoscopic retrieval bags (GeniStrong™ Bag) and morcellation permitted the extraction of the spleen through the 15 mm periumbilical port site. Laparoscopic cholecystectomy was then completed without complication.

**FIGURE 2:**
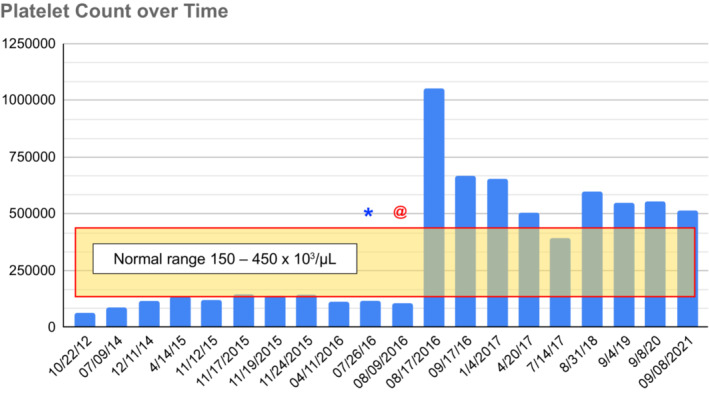
Peripheral platelet counts (normal range 150–450 × 10^3^/μl). *, Date of vaccination administration; @, Date of surgery

Anatomic pathology revealed that the morcellated splenic tissue weighed 602.9 g. Histologically, gallbladder and spleen analyses confirmed chronic cholecystitis with associated cholelithiasis, and excluded malignancies, such as lymphoma; ITP was not confirmed. The patient was discharged home on postoperative day 1. She delivered a healthy infant less than 12 months after surgery. Postoperatively, the patient was followed up closely to monitor the platelet counts and evaluate for hernia development. The 5‐year follow‐up confirmed no postoperative complications, complete resolution of the patient's symptoms, and platelet counts sustained at or just above the normal range, as demonstrated in Figure 2.

## 2 METHODS

We performed a retrospective chart review of a patient who underwent LS for massive splenomegaly. We confirmed sustained resolution of the patient's peripheral platelet count over a 5‐year postoperative period. The findings of this case report were supported by a 2‐year retrospective review of the NSQIP, a risk‐adjusted surgical outcomes US hospital database. Trained surgical NSQIP reviewers collect data using an American College of Surgeons (ACS) validated sampling protocol to reduce sampling bias.^6^ The NSQIP collects data on 135 variables, including preoperative factors, intraoperative risk factors, and 30‐day postoperative outcomes.^7^ ACS audits confirm excellent inter‐rater reliability and report an overall disagreement rate of 2% for all assessed program variables.^6^

We analyzed NSQIP data over the 2‐year period (2016–2018) that corresponded to the period of our patient's surgery. We reviewed patient demographics, comorbidities, peri‐operative factors, and 30‐day postoperative outcomes.

Univariate analyses compared the variables between the LS and OS groups. To measure the differences between the LS and OS groups, we applied the *χ* ^2^ and Fisher's exact tests for categorical variables, the Wilcoxon rank‐sum test for nonparametric continuous variables, and the independent‐samples t‐test for parametric continuous variables.

Outcomes of interest included the mean operative time, estimated blood loss, surgical site infection rate, and length of hospital stay. To control for residual bias, we applied generalized estimating equations. We analyzed treatment effects using a multivariate logistic regression model for dichotomous variables and a multivariable linear regression model for continuous variables. Statistical analysis was performed using the IBM SPSS software (version 24.0) and R software (version 3.3.1; R Foundation for Statistical Computing).

## 3 RESULTS

The patient described in our case report underwent successful laparoscopic cholecystectomy for symptomatic cholelithiasis and LS for massive splenomegaly. She has been symptom‐free for the last 5 years, demonstrated normal platelet counts at the 5‐year follow‐up, and successfully carried a non‐complicated pregnancy to term within 1 year of surgery. Our review of the NSQIP database supported our findings. This analysis included 367 patients who underwent splenectomy for ITP. LS and OS were performed in 325 (89%) and 42 (11%) patients, respectively. Patients were predominantly female (60%). The average age of patients undergoing LS and OS was 50 and 54 years, respectively. Diabetes was more common in patients undergoing OS (33%) than in those undergoing LS (14%). Chronic obstructive pulmonary disease was more common in patients undergoing OS (10%) than in those undergoing LS (2%). No patients who underwent LS were on dialysis, whereas 7% of patients who underwent OS were on dialysis.

The mean operative times were 120 ± 60 min for LS and 122 ± 46 min for OS (*p* = 0.873). Intraoperative transfusion was required in 1.5% of patients who underwent LS versus 14% of patients who underwent OS. There were no superficial surgical site infections (SSI) in patients who underwent LS; 5% of patients who underwent OS developed an SSI. One percent of patients who underwent LS developed an organ space SSI, compared with 7% of patients undergoing OS. Postoperative blood transfusion was required in 6% and 21% of patients who underwent LS and OS, respectively. One percent of patients who underwent LS developed sepsis compared to 5% of patients who underwent OS. Length of hospital stay was 2 days [range: 1, 4] for patients who underwent LS and 6 days [range: 3, 13] (*p* < 0.001) for patients who underwent OS. Table 1 compares the results of our 2022 NSQIP analysis to a 2021 meta‐analysis and a 2013 NSQIP analysis.^1^, ^2^

**TABLE 1:** ITP surgical approach comparison studies

Study	Our 2022 NSQIP Analysis		2021 Meta‐analysis	2013 NSQIP Analysis	
Surgical approach	OS	LS	Comparison of LS to OS	OS	LS
Operative time	122 ± 46 min (*p* = 0.873)	120 ± 60 min	LS longer than OS with WMD in operating time of 49.33 min	100 min	106 min
Blood loss and transfusion data	Intraoperative transfusion in 14% (*p* < 0.001) Postoperative transfusion in 21% (*p* = 0.025)	Intraoperative transfusion in 1.5% Postoperative transfusion in 5%	Less EBL for LS than OS with WMD of −172.59 ml	8.9% w/bleeding or transfusion	2.8% w/bleeding or transfusion
Length of hospital stay	6 days [3, 13] (*p* < 0.001)	2 days [1, 4]	Length of stay of LS is less than OS with WMD ‐4.68 days	6 days	3 days
Surgical site infection	4.8% (superficial) 7% (organ space) (*p* = 0.019)	0% (superficial) 0.6% (organ space)	No significant difference in wound infection between LS (4.6%) and OS (7.1%) *p* = 0.34	1.7% (superficial) 2.2% (organ space) 0.1% (deep incisional)	1% (superficial) 0.85% (organ space) 1.1% (deep incisional)

*Note*: Table 1 compares our data (2022 NSQIP) to the 2021 meta‐analysis (Zhu et al.^2^), and a prior 2013 NSQIP study (Ahad et al.^1^).

Abbreviations: EBL, estimated blood loss; LS, laparoscopic splenectomy; NSQIP, National Surgical Quality Improvement Program; OS, open splenectomy; WMD, weighted mean difference.

## 4 DISCUSSION

We report the 5‐year follow‐up of a patient who underwent combined laparoscopic cholecystectomy and splenectomy for severe symptomatic cholelithiasis, massive splenomegaly, and a planned pregnancy. There were no complications, and the patient remained symptom‐free at the 5‐year follow‐up.

The importance of obtaining a specific diagnosis for patients with idiopathic massive splenomegaly has recently been addressed. Naples et al.^8^ published a 17‐year retrospective review of 68 patients who underwent splenectomy for massive splenomegaly. A definitive diagnosis was determined in 65% of patients following splenectomy. Half of the definitive diagnoses were malignant conditions, with splenic marginal‐zone lymphoma being the most common diagnosis.

The optimal surgical approach for patients undergoing splenectomy in the setting of massive splenomegaly remains controversial. Shin et al.^9^ identified no difference in the complication rates for patients undergoing LS versus OS in a 20‐year retrospective review of 75 patients who underwent splenectomy for massive splenomegaly. The splenectomy procedures were completed laparoscopically in 21%, converted from laparoscopic to open surgery in 12%, and performed as open surgery in 67% of patients. Casaccia et al.^10^ recently reported favorable outcomes in a retrospective analysis of 65 patients undergoing LS versus OS over a 21‐year period. There were 24 patients in the LS group and 41 patients in the OS group. The study demonstrated significantly decreased operative time, estimated blood loss, and length of hospital stay in patients undergoing LS compared with OS.

Significant advances in medical therapies and surgical techniques have been made since the 2013 NSQIP analysis was published. First‐line medical treatment options for ITP include corticosteroids, intravenous immunoglobulin, and anti‐D globulin. Patients who have an inadequate response to primary treatment may consider second‐line therapies, such as rituximab or thrombopoietin receptor agonists, or may opt for splenectomy.^11^ Several new techniques for LS have recently emerged. An example is the active exposure of the pancreatic tail to avoid disruption of the splenic artery, which could result in hemorrhage, in rare cases.^12^ Some surgeons advocate for single‐port laparoscopy as it provides a larger incision for spleen extraction.^13^, ^14^ Newer surgical energy devices, including the Valley Lab LigaSure vessel‐sealing system and Ethicon Harmonic Scalpel, reduce blood loss and operative time.^15^, ^16^ Three investigators have reported a reduced number of bleeding complications when preoperative splenic artery embolization was performed.^17^, ^18^, ^19^ Elective splenectomy allows vaccination to be completed at least 2 weeks preoperatively; if vaccination has not been performed preoperatively, an 8‐week interval is recommended prior to postoperative vaccination of the patient.^20^

Prior research suggests that patients can have a sustained response to either medical therapy or splenectomy, though studies suggest a higher percentage of surgical patients have a sustained response compared to patients who receive medical therapy for ITP. A randomized study demonstrated a sustained response to corticosteroids (platelet count greater than 30 × 10^9^/L for 6 consecutive months) in 41.2% and 40% of patients receiving prednisone and dexamethasone, respectively.^21^ A meta‐analysis of patient response to splenectomy demonstrated that the median rate of complete response (platelet count greater than 100 × 10^9^/L) was 67%.^22^

In prior comparisons of LS and OS, multiple authors have documented longer operative times for LS than for OS. However, the differences are minimal.^1^ Our analysis of NSQIP data demonstrated that it took an average of 2 min longer to perform OS than LS. A 2021 meta‐analysis comparing LS and OS for ITP demonstrated that it took approximately 49 min longer to perform LS compared with OS.^2^ This meta‐analysis included several early studies when LS was less established as a surgical approach.

Several studies have documented a shorter length of hospital stay for LS than for OS. A 2013 NSQIP study demonstrated an average of a 3‐day hospital stay for LS and a 6‐day hospital stay for OS.^1^ A 2021 meta‐analysis demonstrated a reduction of 4.7 days in the length of hospital stay for patients who underwent LS compared with those who underwent OS.^2^ Our patient was discharged on postoperative day 1; our analysis of NSQIP data confirmed an average length of hospital stay of 2 days for LS and 6 days for OS.

Our patient developed no complications or SSI. Previous investigators have demonstrated no differences in the low SSI rates between LS and OS. The 2021 meta‐analysis comparing LS to OS reported SSI rates of 4.6% and 7.1%, respectively; these differences were not significant (OR: 0.65, 95% CI: 0.26–1.59, *p* = 0.34).^2^ The 2013 NSQIP study demonstrated that 1% of patients who underwent LS developed a superficial SSI compared with 2% of patients who underwent OS. The risk of organ space SSI was similarly low; less than 1% of patients in the LS group developed an organ space SSI compared with 2% of patients in the OS group.^1^ Our analysis demonstrated that LS was associated with a lower risk of both superficial (LS, 0%; OS, 5%) and deep organ space SSI (LS, 1%; OS, 7%) compared with OS.

Other investigators have reported lower estimated blood losses (EBL) during LS compared to OS.^1^, ^2^ Our analysis demonstrated that 1.5% of patients who underwent LS required preoperative transfusion, compared to 14% of patients who underwent OS. Postoperatively, 5.5% of patients who underwent LS required transfusion, compared to 21% of patients who underwent OS. The 2013 NSQIP study reported that 5.5% of patients who underwent LS had significant bleeding or required transfusion, compared to 9% of patients who underwent OS.^1^ The 2021 meta‐analysis reported that the EBL was 173 ml less for patients who underwent LS compared to those who underwent OS.^2^ Cavaliere demonstrated a significant reduction in blood loss associated with robotic splenectomy (100 ml) when compared to LS (350 ml).^23^ The reduced volume of blood loss, hospital length of stay, and SSI rates associated with LS compared to OS were likely secondary to the minimally invasive surgical techniques used; however, case selection, in nonrandomized case series, may bias the observed benefits of LS.

Our patient demonstrated an excellent long‐term response to splenectomy, and other studies have reported a robust long‐term response to LS and OS for thrombocytopenia. Qu et al.^24^ reported no significant difference in relapse‐free survival between LS and OS, with a mean follow‐up of 36 months for LS (86% sustained complete remission) and 46 months for OS (91% sustained complete remission). Similarly, Tada et al.^25^ reported that 90% of patients who underwent OS achieved an initial complete or partial remission, compared with 77% of patients who underwent LS; however, this difference was not statistically significant. Navez and Zheng^26^, ^27^ had limited success predicting favorable outcomes using demographic and laboratory data. Istl and Nyilas^28^, ^29^ were able to predict favorable surgical outcomes based on corticosteroid therapy responses. Splenectomy for patients with thrombocytopenia can often be avoided as patient outcomes associated with medical therapy continue to improve. However, splenectomy remains indicated for selected patients with massive splenomegaly when the risk of life‐threatening splenic rupture exceeds the risk of surgery or when the diagnosis of lymphoma should be excluded, particularly in patients with elevated CA 19‐9 and CA 125 levels.

### 4.1 Strengths and limitations

This case report and NSQIP data analysis had limitations.

In our case report, we present the unique case of a patient with symptomatic cholelithiasis, massive splenomegaly, thrombocytopenia, and a planned pregnancy. The generalizability of our case report is limited, as patients rarely present with all four concerns. In addition, we could not confirm nor exclude ITP clinically or pathologically, although the thrombocytopenia resolved post‐splenectomy. The NSQIP analysis, is limited by possible confounding variables, such as surgeon experience and differences in preoperative patient characteristics, that may have impacted our data analysis.

Although trained clinical reviewers collect data for NSQIP, coding errors may also introduce bias within the database.

Despite these limitations, our case report and analysis have several strengths. Our case report demonstrates the benefit of a tailored surgical approach for a patient's unique surgical needs.

Previous studies have demonstrated the safety of combined laparoscopic cholecystectomy and splenectomy. We confirmed excellent clinical outcomes for our patient, immediately postoperatively and at 5‐years follow‐up. The NSQIP data support the efficacy and safety of LS. The NSQIP samples a large patient population at several US hospitals. In addition, our multivariate statistical analysis improved our ability to account for covariates, and we further controlled for confounding variables by adjusting for residual bias.

## 5 CONCLUSION

Our patient provided an important and unique combination of concerns, including severe symptomatic cholelithiasis, massive splenomegaly, and a planned pregnancy. A tailored surgical approach was performed to address all issues. The 5‐year follow‐up demonstrated an excellent patient outcome. Most studies that have compared LS to OS have determined that LS has significant advantages. Our supplemental NSQIP analysis supports that LS is a safe, efficient, and effective approach.

## AUTHOR CONTRIBUTIONS

Alexandra Bishop and Eric Krohn prepared the manuscript. Victor Vakayil and Kyle Pribyl completed the data acquisition and analysis. Mark T. Reding provided clinical management and edited the manuscript. Christopher Tignanelli edited the manuscript. James Harmon provided clinical management and prepared the manuscript.

## CONFLICT OF INTEREST

No conflicts of interest to report.

## FUNDING STATEMENT

No funding was received for this study.

## ETHICAL APPROVAL

The University of Minnesota IRB does not require IRB approval for case reports or de‐identified NSQIP data analysis.

## CONSENT

Written informed consent was obtained from the patient to publish this report, in accordance with the journal's patient consent policy.

## PERMISSION TO REPRODUCE MATERIAL FROM OTHER SOURCES

No material has been reproduced in this manuscript.

## CLINICAL TRIAL REGISTRATION

This is not a clinical trial.

## Data Availability

The data that support the findings of this study are available from the National Surgical Quality Improvement Program (NSQIP). Restrictions apply to the availability of the data, which were used under license for this study. Data are available from the authors with the permission from the NSQIP.
